# Erchen Decoction Offers Health Benefits in Regulating Obesity: Component Analysis, Network Pharmacology, and Experiment Verification

**DOI:** 10.1002/fsn3.70592

**Published:** 2025-08-05

**Authors:** Longfei Zhang, Xiaoxiao Liu, Mingze Xu, Yang Liu, Jiahao Yang, Wenqiong Wang, Hengxian Qu, Tianzhu Guan, Dawei Chen, Ruixia Gu

**Affiliations:** ^1^ College of Food Science and Technology Yangzhou University Yangzhou China; ^2^ Jiangsu Provincial Key Laboratory for Probiotics and Dairy Deep Processing Yangzhou China

**Keywords:** component analysis, Erchen Decoction, molecular docking, network pharmacology, obesity

## Abstract

Erchen Decoction (ECT) is composed of six plants and fruits, with potential for treating metabolic diseases. However, its regulatory effects and material basis on simple obesity have not been reported. In this study, we predicted potential targets and pathways of ECT intervention on simple obesity through component analysis and network pharmacology. Subsequently, the predicted targets and regulatory pathways were validated via animal experiments. A total of 93 compounds were identified in ECT and 221 overlapping potential targets were identified between ECT and obesity. KEGG enrichment analysis revealed that the regulatory pathways of ECT may involve lipid metabolism, oxidative stress regulation, and immune modulation. Molecular docking experiments demonstrated that the main components of ECT, including Sachaliside, Naringenin, Liquiritigenin, maclurin, and Rhapontigenin, exhibited strong binding affinities with oxidative stress and immune markers. Animal experiments demonstrated that ECT has significant potential for weight loss and lipid‐lowering effects, especially mitigating hepatic oxidative stress and systemic inflammation through the LPS‐TLR4/MyD88/NF‐κB signaling pathway. This study provides fundamental data for the further development of ECT as a potential functional food for regulating simple obesity.

## Graphical abstract

This study explored the potential of Erchen Decoction (ECT) in treating simple obesity by integrating network pharmacology, molecular docking, and animal experiments. ECT was found to regulate lipid metabolism, oxidative stress, and immune responses, with key compounds exhibiting strong binding to oxidative stress and immune markers. Animal experiments confirmed its significant weight‐loss and lipid‐lowering effects, primarily by mitigating hepatic oxidative stress and systemic inflammation via the LPS‐TLR4/MyD88/NF‐κB pathway, supporting its potential as a functional food for obesity management.

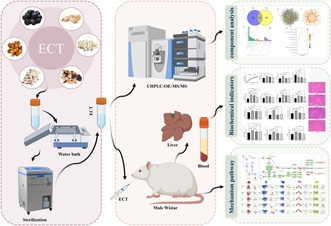

**Abbreviations**
3D: 3‐dimensional
AKT1: serine and threonine kinase AKT1
ALB: serum albumin
ALT: alanine aminotransferase
AST: aspartate aminotransferase
CASP3: caspase‐3
CASP3: caspase‐3
ECT: Erchen Decoction
EGFR: epidermal growth factor receptor
ESR1: estrogen receptor 1
GAPDH: glyceraldehyde‐3‐phosphate dehydrogenase
GLU: glucose
glyLDL: glycosylated low density lipoprotein
GSH: glutathione
GSH‐Px: glutathione peroxidase
HDL‐C: high‐density lipoprotein cholesterol
HIF1A: hypoxia‐inducible factor‐1α
IL‐6: interleukin 6
JZLKL: jiangzhiling granules
LDL‐C: low‐density lipoprotein cholesterol
LPS: lipopolysaccharide
MDA: malondialdehyde
mmLDL: minimally modified low density lipoprotein
NF‐κB: nuclear factor kappa‐B
PPARG: peroxisome proliferator‐activated receptor γ
PPI: protein–protein interaction
ROS: reactive oxygen species
SOD: guperoxide dismutase
Src: tyrosine‐protein kinase src
TC: total cholesterol
TG: triglyceride
TNF‐α: tumor necrosis factor

## 1 Introduction

Obesity is a complex metabolic disorder influenced by multiple factors, including genetic and environmental conditions, and is characterized by excessive fat accumulation in the body. This condition leads to a variety of pathological states. Currently, the global population of obese individuals exceeds 1 billion, making obesity a critical public health issue worldwide. Prolonged obesity can result in chronic inflammation, insulin resistance, and hyperglycemia. It serves as a risk factor for various chronic diseases, such as hypertension, diabetes, hyperlipidemia, and non‐alcoholic fatty liver disease (Perdomo et al. 2023). Currently, traditional treatments such as orlistat (one of the FDA‐approved lipid‐lowering drugs) control obesity by inhibiting lipid metabolism. However, the drug is often associated with various side effects, including gastrointestinal discomfort.

Many plant and fruit combinations can provide a variety of health benefits. ECT, a traditional Chinese prescription composed of six types of plants and fruits with a long history of use, is particularly noteworthy. As a fundamental formula for treating phlegm‐dampness, it is effective in drying dampness, resolving phlegm, regulating Qi, and harmonizing the middle burner (Chen et al. 2023). Modern pharmacological research has demonstrated that ECT has a beneficial regulatory effect on metabolic conditions, including diabetes, hyperlipidemia, atherosclerosis, and fatty liver disease (Li et al. 2023; Zhang et al. 2023; Zhao et al. 2021). According to the Formulary of Peaceful Benevolent Dispensary, the main ingredients of ECT were shown in Table 1. Previous studies showed that ECT could alleviate the progression of NAFLD by inhibiting lipid accumulation (Deng et al. 2024) and could alleviate obesity‐related hepatic steatosis via modulating gut microbiota‐driven butyric acid contents and promoting fatty acid β‐oxidation (Zhang et al. 2023). However, the regulatory effects of ECT on simple obesity and the underlying mechanisms of action, particularly from the perspectives of network pharmacology and molecular docking, remain underexplored.

**TABLE 1:** Composition information of ECT.

Chinese name	Figure	Botanical name	Part used	Amount used
Fabanxia	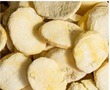	Licorice‐cured rhizome of *Pinellia ternata* (Thunb.) Makino (Family: Araceae)	Tuber	9 g
Fuling	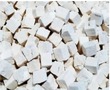	Sclerotium of Poria cocos (Schw.) Wolf (Family: Polyporaceae)	Sclerotia	6 g
Chenpi	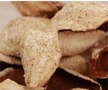	*Citrus reticulata* Blanco and its cultivated varieties (Family: rutaceae)	Mature pericarp	9 g
Shengjiang	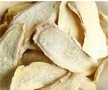	Fresh rhizome of *Zingiber officinale* Roscoe (Family: Zingiberaceae Martinov)	Rhizome	3 g
Zhigancao	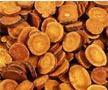	Dried root and rhizome of Glycyrrhiza uralensis Fisch (Family: Fabaceae Lindl)	Root and rhizome	3 g
Wumei	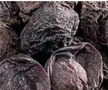	Fruit of *Prunus mume* (Sieb.) Sieb. et Zucc. (Family: Rosaceae)	Near‐mature fruit	1 piece

Network molecular pharmacology is a discipline dedicated to exploring the interactions between drugs and molecular networks within biological systems. By analyzing these networks, key signaling nodes are identified, enabling the study of multi‐target drug molecules. This approach also allows for the visualization of compound‐target protein/gene‐disease relationships, shedding light on the potential regulatory pathways of active compounds within individual plant/fruit or plant/fruit prescriptions for specific diseases (Wu et al. 2018). Currently, network pharmacology analyses of classical prescriptions such as Wumei Wan (Duan et al. 2023), Sijunzi Decoction (Shang et al. 2023), Qinlian Hongqu Decoction (Zhang et al. 2024), and Huai Hua San (Liu et al. 2021) have been conducted. These results suggested that network molecular pharmacology can be used as an effective tool to study the potential mechanism of ECT intervention in obesity.

The aim of this study was to investigate the effects of ECT on simple obesity and explore the underlying mechanisms of its active compounds. UHPLC‐OE‐MS/MS technology, combined with a network pharmacology approach, was utilized to elucidate the substance basis of ECT on anti‐obesity. The weight loss potential of ECT was evaluated using a high‐fat diet‐induced obese rat model, while molecular docking techniques were used to explore possible mechanisms of action.

## 2 Materials and Methods

### 2.1 Chemicals, Reagents and Materials

TG, TC, MDA, and GSH kits, as well as GSH‐Px and SOD enzyme activity kits were purchased from Nanjing Jiancheng Bioengineering Research Institute. ELISA kits for NF‐κB, IL‐6, and TNF‐α were purchased from Shanghai Hualan Chemical Technology Co. Ltd. All primers were designed by Sangon Biotech (Shanghai) Co. Ltd.

All six herbal ingredients of ECT in Table 1 were purchased from Anzhutang Biotechnology Co. Ltd. in Lu'an City, Anhui Province. All plants were identified by Ying Yao, chief pharmacist of Yangzhou University, and the voucher specimens were kept in Jiangsu Provincial Key Laboratory for Probotics and Dairy Deep Processing.

### 2.2 Preparation of ECT

The dried materials were fully crushed and passed through an 80‐mesh sieve for later use. The mixture was then soaked at a material‐to‐water ratio of 1:15 (w/w) at 85°C for 2 h. Following sterilization at 121°C for 15 min, the preparation was set aside for subsequent use.

### 2.3 Determination of the Main Components of ECT

#### 2.3.1 Pretreatment Conditions

To identify the main chemical constituents in the ECT water extract, the extract was centrifuged at 12,000 rpm for 5 min. The resulting supernatant was then filtered through a 0.45 μm aqueous membrane (Beekman, Jiangsu, China) and set aside for further analysis.

#### 2.3.2 Mass Spectrometry Conditions

A Dionex 3000 Ultimate UHPLC system coupled with a Q‐Exactive Orbitrap high‐resolution mass spectrometer (Thermo Fisher, MA, USA) was employed for the analysis. Chromatographic separation was carried out on an Acquity UPLC BEH C18 column (4.6 × 100 mm, 1.8 μm) at a column temperature of 40°C. The mobile phase consisted of 0.1% formic acid in water (A) and acetonitrile (B), with a flow rate of 0.4 mL/min. The gradient elution program was as follows: 0–5 min, phase B increased linearly from 3% to 55%; 5–15 min, phase B increased from 55% to 80%; 15–19 min, phase B increased from 80% to 100%; 19–20 min, phase B decreased from 100% to 3%, followed by a 3‐min rebalancing of the system.

Mass spectrometry analysis was performed in both positive and negative ion modes with the following settings: the sheath gas flow rate was 55 arb, and the auxiliary gas flow rate was 15 arb. Spray voltages were 3.5 kV in positive mode and 3.8 kV in negative mode, respectively. The mass scanning range was set to 60–1000 m/z. The capillary temperature was maintained at 350°C, and the probe heater temperature at 300°C.

#### 2.3.3 Substance Screening

The raw data was converted into mzXML format by ProteoWizard software, and metabolite identification was carried out using the collaboratively written R package. The database used is BiotreeDB (V3.0), and substances were determined based on retention time, peak area size (> 0.1%), and secondary mass spectrum score.

### 2.4 Network Pharmacology Analysis

#### 2.4.1 Determination of the Target of Action of Components in ECT

The targets of the bioactive components in ECT were obtained by searching for the identified substances in the STITCH (http://stitch.embl.de/) and Swiss Target Prediction databases (http://www.swisstargetprediction.ch/). The targets were then standardized using the UniProt database (https://www.uniprot.org), with the species and status set to “human” and “reviewed” respectively.

#### 2.4.2 Screening Possible Targets for ECT Intervention in Obesity

The presumed target proteins for obesity were identified by examining the CTD database (http://ctdbase.org/) and OMIM (https://omim.org/). The intersection of the two sets of target proteins was taken as the final target proteins.

#### 2.4.3 Construction of the PPI Network and Pathway Prediction for ECT in the Treatment of Obesity

KEGG functional enrichment analysis and visualization were performed using Metascape (https://metascape.org/). The interaction network of key ECT‐Obesity targets was constructed using Cytoscape 3.7.1.

### 2.5 Molecular Docking

Molecular docking technology was employed to study the intermolecular interaction between core target proteins and the primary active components of ECT in 3D space. The top 15 core components were docked with SOD, GSH‐Px, IL‐6, TNF‐α, and NF‐κB. The 3D structures of the target proteins were obtained in PDB format from the RCSB PDB database (http://www.rcsb.org/), while the structures of the active components of ECT were retrieved in mol2 format from the TCMSP database (https://old.tcmsp‐e.com/).

### 2.6 Animal Experimental Verification of ECT Regulating Obesity

#### 2.6.1 Animal Experiments

Male Wistar rats (4 weeks old, 140–160 g) were obtained from the Medical Animal Experiment Center of Yangzhou University (Yangzhou, License No.: SCXK2016‐0006). The environmental conditions were maintained at a temperature of 26°C ± 1°C and a humidity of 55% ± 5%, with a 12‐h light/dark cycle. The rats had free access to water and food. The standard feed (product code: XT93M) and high‐fat feed (product code: XTHF45) were supplied by Jiangsu Xietong Pharmaceutical Bioengineering Co. Ltd.

**FIGURE 1:**
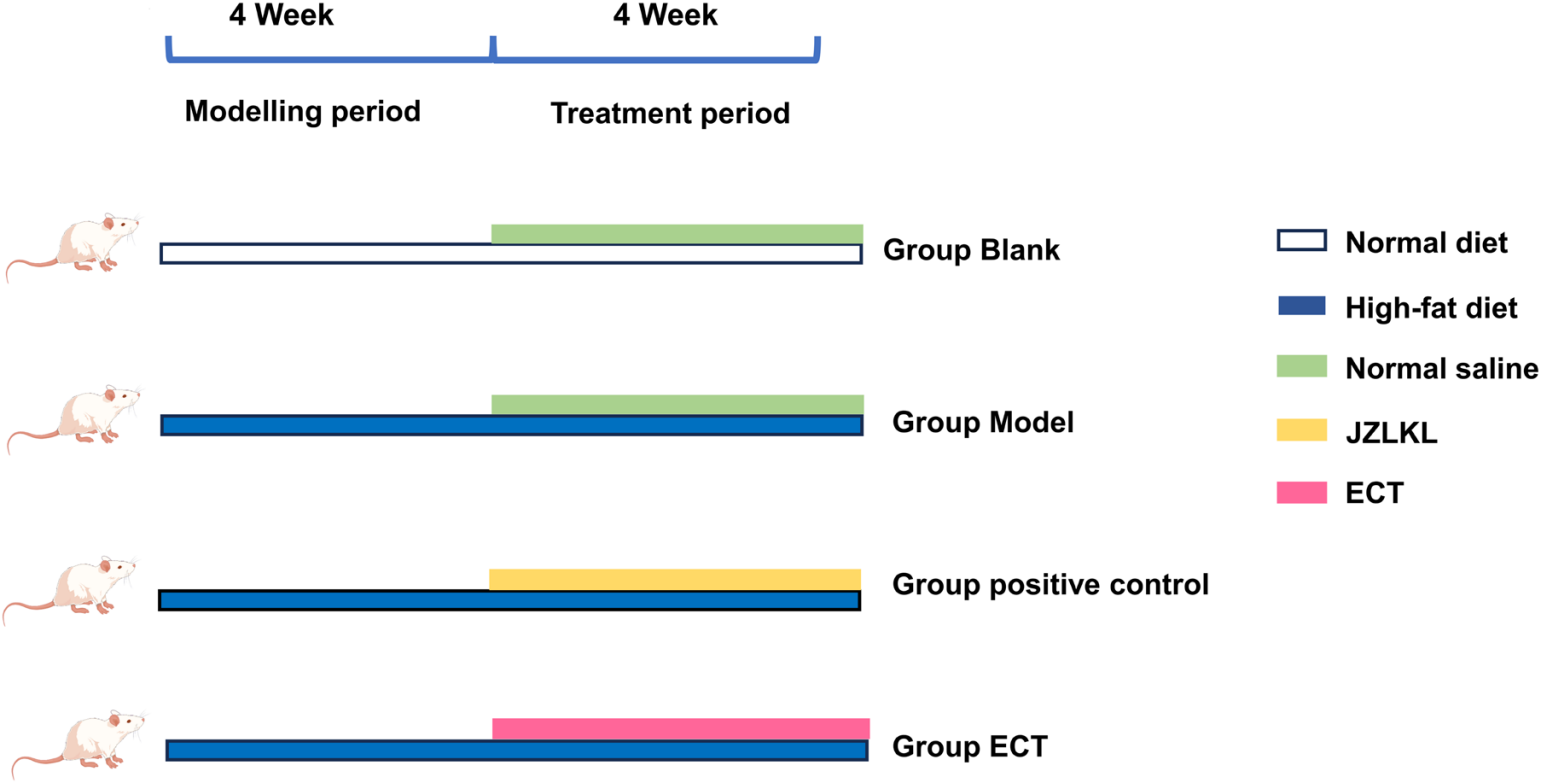
Animal experiment schematic.

After an acclimatization period, the rats were randomly divided into four groups (*n* = 8), as shown in Table 2. Except for the blank group, all other groups were fed with a high‐fat diet for a modeling period of 4 weeks. The animal experimental procedures were reviewed and approved by the Institutional Animal Care and Use Committee (IACUC) of Yangzhou University, and the ethical approval number is 202503249. The body weight was measured and recorded weekly. A body weight increase of more than 10% compared to the blank group confirmed successful model establishment. The experimental design was shown in Figure 1. All animal experiments were conducted in strict accordance with the ARRIVE (Animal Research: Reporting of In Vivo Experiments) guidelines.

**TABLE 2:** Animal experiment grouping.

Group name	Intragastric sample	Feeding amount
Blank	Normal saline	1.8 mL/100 gd^−1^
Model	Normal saline	
Positive control	Jiangzhiling granules	
ECT	ECT	

#### 2.6.2 Basic Indicator Measurement

The rats were weighed weekly, and a body weight change chart was generated. Daily food intake was also recorded. After the intervention, the rats were weighed, and their body lengths were measured. The food effect ratio was calculated using the following formula: Food effect ratio (%) = (body weight gain (mg) / total food intake (g)) × 100. Lee's index was calculated according to the following formula: Lee's index = Weight^(1/3)/Length.

Following anesthesia, blood samples were collected via enucleation of the eyeballs. The blood was then centrifuged (3000 × g, 10 min, 4°C) to collect serum samples. After the rats were sacrificed, the liver, epididymis fat, and subcutaneous white fat were dissected and weighed. The tissue index was calculated using the formula: Tissue index (%) = tissue mass (g)/body weight (g) × 100.

#### 2.6.3 Determination of Biochemical Indexes in Rats

Serum levels of GLU, HDL‐C, LDL‐C, TG, TC, ALT, and AST were measured using a 7020 automatic biochemical analyzer (Hitachi, Tokyo, Japan).

The liver was accurately weighed, and physiological saline was added at a 1:10 ratio. The mixture was homogenized using a glass homogenizer. The contents of MDA and GSH, as well as the activities of GSH‐Px and SOD enzymes, were measured in the liver homogenate to assess oxidative stress levels in the rats. Additionally, TG and total cholesterol TC levels in the liver homogenate were determined.

#### 2.6.4 Liver Tissue Observation

To evaluate the efficacy of ECT in the treatment of liver injury caused by simple obesity, the histopathological changes of rat livers were observed. Liver specimens were collected, rinsed with PBS, and fixed with 10% formalin. Subsequently, they were transferred to 75% ethanol, and the fixed tissues were embedded in paraffin and cut into 5 μm sections. To observe the cell structure, the sections were stained with a hematoxylin and eosin (HE) staining kit and a periodic acid‐Schiff (PAS, Beijing Sorabio Technology Co. Ltd., Beijing, China) staining kit, respectively.

### 2.7 Total RNA, cDNA Synthesis and Real‐Time PCR

Total RNA was extracted from rat colon tissues across all experimental groups using the MiPure Cell/Tissue miRNA Kit (Vazyme, Nanjing, China), following the manufacturers protocol. Complementary DNA (cDNA) was synthesized using the HiScript Q RT SuperMix for qPCR (+gDNA wiper) (Vazyme, Nanjing, China) to eliminate genomic DNA contamination. The target genes analyzed included NF‐κB (Forward: TCCCACAAGGGGACATTAAGC; Reverse: CAATGGCCTCTGTGTAGCCC), IL‐6 (Forward: TCCGGAGAGGAGACTTCACA; Reverse: CAATGGCCTCTGTGTAGCCC), TNF‐α (Forward: ACTGAATTCCGGGGTGATTG; Reverse: GCTTGGTGGTGTTTGCTACGAC), TLR4 (Forward: TATAAAACATAAGGAGAGGAGG; Reverse: ATAGAAAAAGGGAAAGGAAGGA), MyD88 (Forward: ACCGCATCGAGGAGGACTG; Reverse: CTGTGGGACACTGCTCTCCA), and ACTB (Forward: CCACCATGTACCCAGGCATT; Reverse: CGGACTCATCGTACTCCTGC), with ACTB selected as the internal reference gene.

Quantitative real‐time PCR (qRT‐PCR) was performed using the StepOne/StepOnePlus Real‐Time PCR System (Applied Biosystems, USA) under the following thermal cycling conditions:

- Initial denaturation: 95°C for 30 s.
- Amplification: 40 cycles of 95°C for 10 s and 60°C for 30 s.
- Melt curve analysis: 60°C to 95°C with 1°C increments every 15 s.

Relative gene expression levels were calculated using the 2^−ΔΔCt^ method after verifying the specificity of amplification through melt curve analysis.

### 2.8 Statistical Methods

All statistical analyses were conducted using GraphPad Prism (version 8.0, GraphPad Software, San Diego, USA). Data are presented as mean ± standard deviation (SD). Differences between two groups were assessed using an unpaired two‐tailed Student's *t*‐test. For comparisons among three or more groups, one‐way analysis of variance (ANOVA) was performed, followed by the least significant difference (LSD) post hoc test. A *p*‐value of less than 0.05 was considered statistically significant.

## 3 Results

### 3.1 Identification of Compounds in ECT

The compounds in the aqueous extract of ECT were identified using UHPLC‐OE‐MS/MS technology. The TIC chromatograms in both positive and negative ion modes were shown in Figure S1. By analyzing and comparing the retention times, m/z values, and major MS^2^ fragment ions of the compounds, the results were matched against the BiotreeDB (V3.0) database. Subsequently, applying a threshold of relative peak area > 0.1%, 93 compounds were identified, primarily including polyphenols, flavonoids, terpenes, organic acids, polysaccharides, and others. Detailed information was provided in Table 3. Among them, eight major compounds with a relative peak area > 1% were identified: Citric acid (organic acid), 3‐Methylbutanamine (terpene), Gingerol (phenol), Sarpogrelate (phenol), Malic acid (organic acid), Hesperidin (flavonoid), Sphingosine (organic acid), and Sorbose (sphingolipid).

**TABLE 3:** The compounds in ECT.

No.	Proposed compound	RT/s	Observed m/z	Formula	Content (%)
1	Citric acid	191.0198	26.5	C_2_H_6_OS	7.398886
2	3‐Methylbutanamine	88.1117	358.1	C_5_H_13_N	4.582444
3	Gingerol	293.1755	233.8	C_21_H_34_O_4_	2.805281
4	Sarpogrelate (hydrochloride)	410.1938	245.2	C_24_H_31_NO_6_	2.738222
5	Malic acid	133.0142	25.8	C_4_H_6_O_5_	1.956205
6	Hesperidin	609.1818	181.7	C_28_H_34_O_15_	1.439991
7	Sphingosine	282.2783	310.2	C_18_H_37_NO_2_	1.329257
8	Sorbose	179.056	24	C_6_H_12_O_6_	1.024025
9	Palmitamide	256.2627	308.2	C_16_H_33_NO	0.942157
10	Tetracosanoic acid	367.3573	329.1	C_24_H_48_O_2_	0.870092
11	Choline	104.1066	23.4	C_5_H_14_NO	0.783292
12	7‐hydroxy‐2‐[4‐[(2S,3R,4S,5S,6R)‐3,4,5‐trihydroxy‐6‐(hydroxymethyl)tetrahydropyran‐2‐yl]oxyphenyl]chroman‐4‐one	417.1185	179	C_21_H_22_O_9_	0.779071
13	Magnolioside	353.0871	149.7	C_16_H_18_O_9_	0.761963
14	7‐(2‐hydroxypropan‐2‐yl)‐1,4a‐dimethyl‐2,3,4,5,6,7,8,8a‐octahydronaphthalen‐1‐ol	258.2419	281	C_15_H_28_O_2_	0.696319
15	Heptamethoxyflavone	433.1478	224.5	C_22_H_24_O_9_	0.646161
16	(R)‐10‐Hydroxystearate	318.2993	229	C_18_H_35_O_3_^−^	0.60567
17	Palmitic acid	255.2327	321.7	C_16_H_32_O_2_	0.602857
18	Turanose	341.108	24.7	C_12_H_22_O_11_	0.592585
19	DEHP	391.2833	323	C_24_H_38_O_4_	0.557622
20	Linoleamide	280.2627	299.2	C_18_H_33_NO	0.547922
21	Stearic acid	283.2639	335.7	C_18_H_36_O_2_	0.535359
22	Shogaol	277.1789	234.1	C_19_H_28_O_3_	0.432224
23	Hydroxyferulic acid	209.0454	78.1	C_10_H_10_O_5_	0.423501
24	Eduline	266.1228	24.8	C_17_H_15_NO_2_	0.419912
25	Hexacosanoic acid	395.3887	342.6	C_26_H_52_O_2_	0.414257
26	Isoferulic acid	193.0504	179.8	C_10_H_10_O_4_	0.405155
27	FA 18:1 + 3O	329.2331	231.4	C_18_H_34_O_5_	0.400217
28	Tricosanoic acid	353.3417	282.3	C_23_H_46_O_2_	0.390756
29	Liquiritigenin	255.0662	202.7	C_15_H_12_O_4_	0.358121
30	Adenosine	268.1036	47.6	C_10_H_13_N_5_O_4_	0.349936
31	methyl (E)‐8‐(4‐hydroxy‐6‐methoxy‐7‐methyl‐3‐oxo‐1H‐2‐benzofuran‐5‐yl)‐2,6‐dimethyloct‐6‐enoate	375.1838	248.7	C_21_H_28_O_6_	0.337264
32	Diammonium Glycyrrhizinate	823.4085	245.5	C_42_H_62_O_16_	0.320552
33	2‐methylcitrate	205.0352	28.8	C_7_H_10_O_7_	0.320134
34	(2S,3R,4R,5R,6S)‐2‐[(2R,3R,4S,5R,6R)‐2‐[2‐(3,4‐dihydroxyphenyl)ethoxy]‐3,5‐dihydroxy‐6‐(hydroxymethyl)oxan‐4‐yl]oxy‐6‐methyloxane‐3,4,5‐triol	461.1663	154.9	C_20_H_30_O_12_	0.316335
35	Lauric diethanolamide	288.2525	267.4	C_16_H_33_NO_3_	0.313671
36	7‐Methylxanthine	165.0404	23.6	C_6_H_6_N_4_O_2_	0.309023
37	2‐[(4‐hydroxyphenyl)methyl]propanedioic acid	209.0454	57.5	C_10_H_10_O_5_	0.303355
38	Pinolenic_Acid	277.217	303.1	C_18_H_30_O_2_	0.301532
39	Feretoside	405.1432	222.1	C_17_H_24_O_11_	0.299767
40	Pelargonic acid	157.1233	262	C_9_H_18_O_2_	0.283309
41	5‐Hydroxytryptophan	203.076	19.9	C_11_H_12_N_2_O_3_	0.280068
42	Ononin	431.1325	193.5	C_22_H_22_O_9_	0.275206
43	Flavone base +3O, 1MeO, C‐Hex‐Hex	623.1616	163.2	C_28_H_32_O_16_	0.272666
44	1‐(2,6‐dihydroxyphenyl)‐9‐phenyl‐nonan‐1‐one	325.1841	326.2	C_21_H_26_O_3_	0.249111
45	Levonordefrin	166.086	40.6	C_9_H_13_NO_3_	0.248086
46	5,7‐dihydroxy‐2‐(4‐hydroxyphenyl)‐6,8‐bis[3,4,5‐trihydroxy‐6‐(hydroxymethyl)tetrahydropyran‐2‐yl]chromen‐4‐one	595.1638	163.5	C_27_H_30_O_15_	0.243418
47	Triptophenolide	311.1685	318.7	C_20_H_24_O_3_	0.229137
48	Dicyclopenta[a,i]phenanthrene‐3a(1H)‐carboxylic acid, 10‐formyloctadecahydro‐9‐hydroxy‐5a,5b,8,8,10a‐pentamethyl‐1‐(1‐methylethenyl)—	453.3351	248.5	C_30_H_46_O_4_	0.22226
49	(9Z)‐Octadecenoic acid	283.2611	309.8	C_18_H_34_O_2_	0.222062
50	Formononetine	267.0661	198.6	C_16_H_12_O_4_	0.218129
51	Glutarylcarnitine	276.1437	27.6	C_12_H_21_NO_6_	0.214935
52	Isocitric acid	173.0091	26.8	C_6_H_8_O_7_	0.212687
53	Complanatuside	625.1741	163.9	C_28_H_32_O_16_	0.212088
54	3‐oxo‐3‐[[(2R,3S,4S,5R,6S)‐3,4,5‐trihydroxy‐6‐[3‐(4‐methoxyphenyl)‐4‐oxochromen‐7‐yl]oxyoxan‐2‐yl]methoxy]propanoic acid	517.1324	198.8	C_25_H_24_O_12_	0.208782
55	Miserotoxin	268.1035	29.5	C_9_H_17_NO_8_	0.206753
56	(10E,15Z)‐9,12,13‐trihydroxyoctadeca‐10,15‐dienoic acid	327.2175	228.8	C_18_H_32_O_5_	0.203134
57	Quinate	191.0548	36	C_7_H_12_O_6_	0.200492
58	Coumarin base +1O, 1MeO, O‐Hex	399.093	147.7	C_16_H_18_O_9_	0.187236
59	3′‐Demethylnobiletin	389.122	214.3	C_20_H_20_O_8_	0.185511
60	GLYCEROL 2‐PHOSPHATE	170.9996	146.7	C_3_H_9_O_6_P	0.184486
61	Azelaic acid	187.0975	197.8	C_9_H_16_O_4_	0.17129
62	(−)‐Maackiain‐3‐O‐glucosyl‐6″‐O‐malonate	531.1143	175.2	C_25_H_24_O_13_	0.166033
63	[(2R,3S,4S,5R,6S)‐6‐[(2S,3R,4S,5R,6R)‐6‐[[(1S,3R,4S,4aR,8aR)‐4‐[(3S)‐3‐[(2S,3R,4R,5R,6R)‐3,4‐dihydroxy‐6‐methyl‐5‐[(2S,3R,4R,5R,6S)‐3,4,5‐trihydroxy‐6‐methyloxan‐2‐yl]oxyoxan‐2‐yl]oxy‐3‐methylpent‐4‐enyl]‐3,4,8,8a‐tetramethyl‐1,2,3,4a,5,6‐hexahydronaphthalen‐1‐yl]oxy]‐4,5‐dihydroxy‐2‐methyloxan‐3‐yl]oxy‐3,4,5‐trihydroxyoxan‐2‐yl]methyl acetate	983.4482	218.6	C_46_H_76_O_20_	0.165838
64	6‐(hydroxymethyl)pyridin‐3‐ol	126.0547	29.1	C_6_H_7_NO_2_	0.165815
65	8‐Gingerol	321.207	255.5	C_19_H_30_O_4_	0.16404
66	5,7‐dihydroxy‐2‐(4‐hydroxyphenyl)‐2,3‐dihydro‐4H‐chromen‐4‐one	273.0813	29.7	C_15_H_12_O_5_	0.162102
67	(2R,3S,4S,5R,6R)‐2‐(hydroxymethyl)‐6‐[(E)‐3‐(4‐hydroxyphenyl)prop‐2‐enoxy]oxane‐3,4,5‐triol	330.1541	25.5	C_15_H_20_O_7_	0.16037
68	maclurin	263.0606	24.1	C_13_H_10_O_6_	0.158475
69	2‐[4‐[3‐[3,4‐dihydroxy‐4‐(hydroxymethyl)tetrahydrofuran‐2‐yl]oxy‐4,5‐dihydroxy‐6‐(hydroxymethyl)tetrahydropyran‐2‐yl]oxyphenyl]‐7‐hydroxy‐chroman‐4‐one	549.1614	192	C_26_H_30_O_13_	0.156532
70	Linoleic acid	279.2327	312.4	C_18_H_32_O_2_	0.156497
71	Naptalam	290.0879	25.4	C_18_H_13_NO_3_	0.153657
72	9‐(Methylsulfinyl)nonanenitrile	224.1124	23.8	C_10_H_19_NOS	0.150352
73	Pyroglutamic acid	128.0353	26.1	C_5_H_7_NO_3_	0.146581
74	Benzoic acid	121.0295	194.1	C_9_H_8_O_3_	0.144584
75	4‐Chlorophenylalanine	200.0468	79.9	C_9_H_10_ClNO_2_	0.140837
76	Aloin	436.1591	179.4	C_21_H_22_O_9_	0.140238
77	Capric acid	171.139	272.6	C_10_H_20_O_2_	0.133288
78	Allamandin	309.0963	150	C_15_H_16_O_7_	0.132486
79	Eudesmin	387.1791	237.1	C_22_H_26_O_6_	0.132014
80	Salicyluric acid	176.0402	150.2	C_9_H_9_NO_4_	0.131633
81	Citiolone	142.0347	33.5	C_6_H_9_NO_2_S	0.126091
82	dodecanamide	200.2003	273.3	C_12_H_25_NO	0.123315
83	Cinnamaldehyde	133.0605	25.9	C_9_H_8_O	0.121305
84	1‐Deoxypebrolide	415.2105	249.3	C_24_H_30_O_6_	0.117348
85	6‐[(2E)‐3,7‐dimethylocta‐2,6‐dienyl]‐7‐hydroxy‐chromen‐2‐one	297.1528	309.6	C_19_H_22_O_3_	0.115401
86	Rhapontigenin	281.0699	25.2	C_15_H_14_O_4_	0.114329
87	Homoveratric acid	195.0663	173.4	C_10_H_12_O_4_	0.113704
88	Methyl 9‐hydroxy‐2,6,6,11,15,18‐hexamethyl‐16‐methylidene‐5,8,14‐trioxo‐10,13‐dioxapentacyclo[10.5.2.02,7.09,18.015,19]nonadeca‐3,11‐diene‐19‐carboxylate	469.1864	211	C_26_H_30_O_8_	0.113456
89	O‐Oxalylhomoserine	192.055	29	C_6_H_9_NO_6_	0.111852
90	Iristectorin B	493.1329	180.2	C_23_H_24_O_12_	0.105602
91	(3R,4S)‐4,6,8‐trihydroxy‐7‐methoxy‐3‐methyl‐isochroman‐1‐one	239.0593	239.9	C_11_H_12_O_6_	0.105545
92	3‐hydroxy‐2‐[(Z)‐oct‐2‐enyl]pentanedioic acid	239.1285	247.9	C_13_H_22_O_5_	0.104248
93	3,9‐Dimethoxypterocarpan	283.0954	137.9	C_17_H_16_O_4_	0.102957

Abbreviations: No., number; RT, remain time.

### 3.2 Network Pharmacology and Pathway Analysis

#### 3.2.1 Screening of Drug Ingredient Targets and Obesity‐Related Therapeutic Targets

The potential targets of ECT components were identified using the STITCH and Swiss Target Prediction databases, and a total of 3444 targets were obtained. By applying a screening criterion of target probability > 0.1, a total of 2444 potential targets were obtained. After deleting duplications, the number of drug ingredient targets obtained was 746. With obesity as the key word, after querying 2 databases and deleting duplicates, a total of 1344 presumed target protein targets were obtained.

#### 3.2.2 Construction of Protein–Protein Interaction Network Map and Screening of Key Targets

**FIGURE 2:**
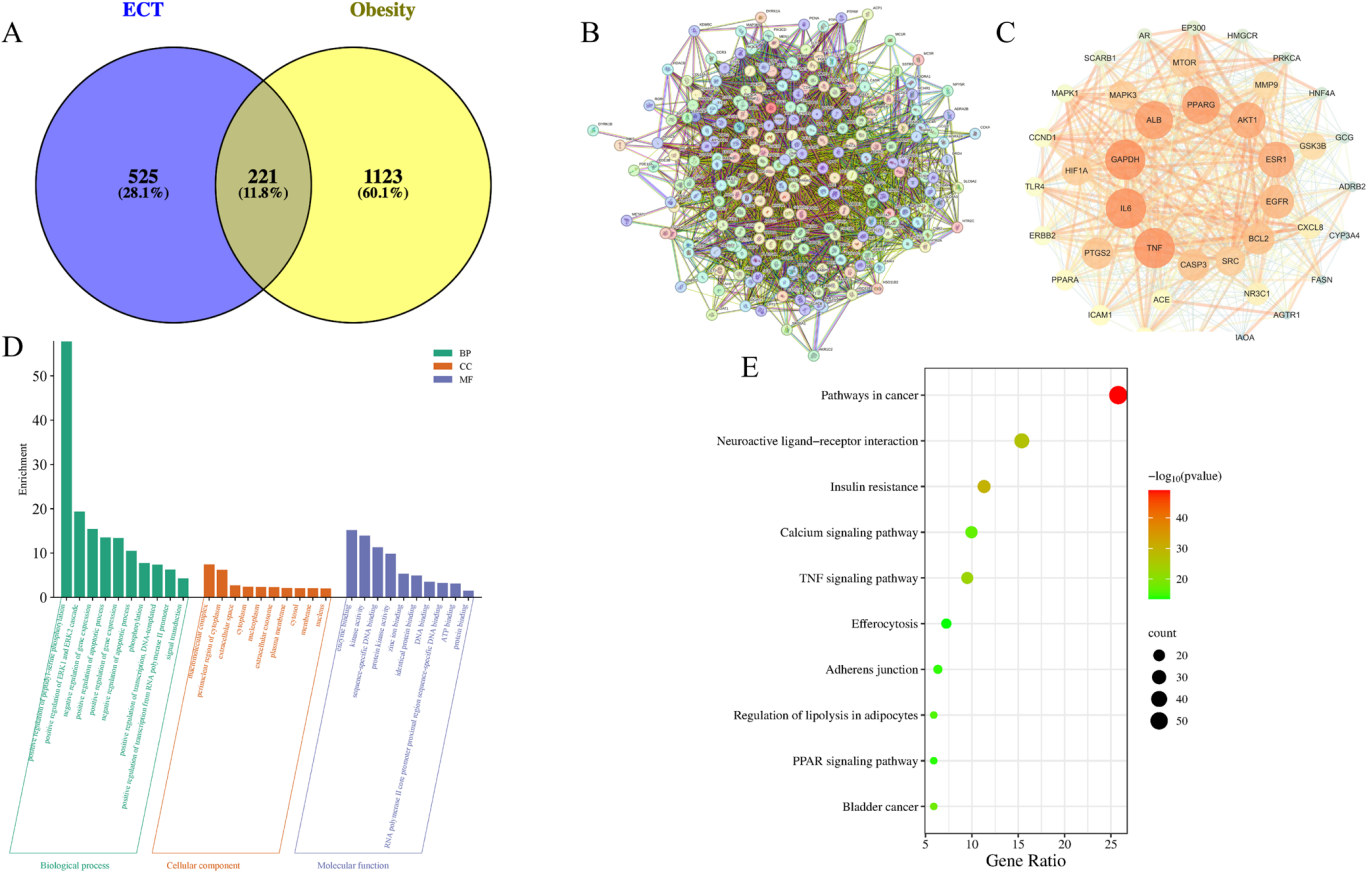
Analysis of proteins potentially affected by ECT treatment. (A) Proteins interacting with components of ECT and proteins known or predicted to be involved in the pathogenesis of obesity. (B) Analysis diagram illustrating the network of target‐protein interactions. (C) Key subnetwork of top nodes analyzed by CytoHubba. (D) Analysis of the GO classification of the same set of proteins. (E) KEGG enrichment results of the interacting proteins represented as a bubble map.

The 746 drug targets of the active components in ECT were compared with the 1344 obesity‐related treatment targets, resulting in 221 overlapping targets (Figure 2A). The 221 targets may be the main targets for ECT intervention in obesity. The intersection of the two target protein sets was used as the final target proteins, and Cytoscape 3.7.1 was employed to construct a PPI network analysis diagram (Figure 2B). After a second round of filtering (betweenness > 205.24, closeness > 0.002, degree > 36.923), a core target network was constructed, consisting of 40 nodes and 593 edges, with an average degree value of 83.35. The interaction data was imported into Cytoscape 3.10 to generate the PPI network (Figure 2C). The targets were ranked in descending order based on their degree values according to network topological characteristics (degree ≥ 95), with the results shown in Table 4. A higher degree value indicates greater importance of the node within the network. Based on the ranking results, 11 key molecules were identified: AKT1, IL6, TNF, ALB, GAPDH, PPARG, SRC, ESR1, EGFR, HIF1A, and CASP3. The calculated average shortest path length, betweenness centrality, and closeness centrality of the network nodes were shown in Table 4.

**TABLE 4:** Topological parameters of the targets.

Name	Degree	Closeness centrality	Betweenness centrality
AKT1	148	0.003425	2929.351
IL6	140	0.003333	2382.494
TNF	139	0.003322	2297.457
ALB	134	0.003257	2098.356
GAPDH	131	0.003236	2018.755
PPARG	131	0.003236	2426.162
SRC	121	0.003135	3388.354
ESR1	106	0.002994	1486.686
EGFR	103	0.002967	833.4906
HIF1A	101	0.00295	583.8365
CASP3	99	0.002933	448.0762

#### 3.2.3 Functional Analysis Based on GO Classification and KEGG Pathway

To further investigate the biological processes (BP), molecular functions (MF), and cellular components (CC) of these key proteins, GO enrichment analysis was performed. A total of 433 pathways were enriched, including 332 for BP, 37 for CC, and 64 for MF (*p* < 0.05). The top 10 GO terms (*p* < 0.05) were selected as significant entries based on *p*‐values (Figure 2D). This GO category analysis indicates that most hubs were involved in processes such as peptidyl‐serine phosphorylation, ERK1 and ERK2 cascade, gene expression, and apoptotic process. Molecular function analysis showed that the proteins were mainly involved in protein binding, kinase activity, sequence‐specific DNA binding, and protein kinase activity. In terms of cellular localization, these proteins were predominantly found in macromolecular complexes, the perinuclear cytoplasm, and other cellular components.

KEGG pathway enrichment analysis indicated that the key targets contribute to 185 different pathways (organization = “has”, *p* < 0.05). Among the pathways enriched based on degree value, the most prominent included Pathways in cancer, Insulin resistance, Neuroactive ligand‐receptor interaction, TNF signaling pathway, Bladder cancer, Calcium signaling pathway, Regulation of lipolysis in adipocytes, Adherens junction, PPAR signaling pathway, and Efferocytosis.

### 3.3 Effect of ECT on Simple Obesity Rats

**FIGURE 3:**
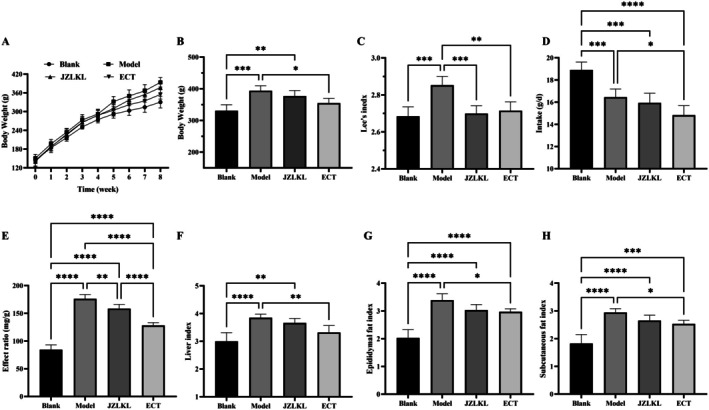
Effect of ECT on the physique of obese rats fed with high‐fat diet. (A) Changes in body weight, (B) body weight at the end of intervention, (C) effect in Lee's index, (D) effect in intake, (E) effect ratio of food, (F) Liver index, (G) Epididymal fat index, and (H) Subcutaneous fat index. *, **, ***, and **** represent significant difference between different groups.

To determine the regulatory effects of ECT on high‐fat diet‐induced obese rats, measurements of the body weight, Lee's index, food intake, food efficiency ratio, liver index, epididymal fat index, and subcutaneous fat index were conducted. The results were shown in Figure 3A–H. ECT demonstrated an ability to mitigate the persistent weight gain associated with the high‐fat diet. By the 8th week, the ECT group exhibited significantly lower weight compared to the model group (*p* < 0.05), with no significant difference compared to the blank group (*p* > 0.05). ECT significantly reduced the Lee's index, decreased the high‐fat diet intake of the rats, and lowered the feed conversion rate (*p* < 0.05) (Figure 3C–E). Additionally, after 4 weeks of ECT intervention, the liver index, epididymal fat index, and subcutaneous white fat index were significantly reduced, with no significant difference in the regulatory effects compared to those of JZLKL granules (*p* > 0.05). These results indicated that ECT can regulate simple obesity induced by a high‐fat diet, with effects such as reducing body weight, regulating food intake, inhibiting food conversion efficiency, reducing liver enlargement, and decreasing fat accumulation.

### 3.4 The Regulatory Effects of ECT on Blood Lipids and Liver Function in Rats

**FIGURE 4:**
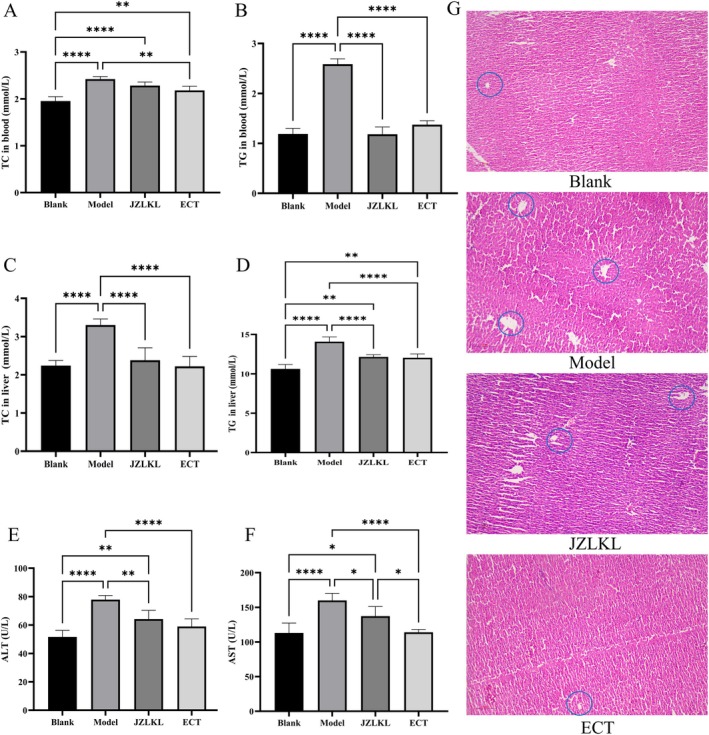
Effect of ECT on serum and liver biochemistry parameters. (A) TC in blood, (B) TG in blood, (C) TC in liver, (D) TG in liver, (E) ALT in blood, (F) AST in blood, and (G) Liver sections of different groups (×100); blue circle represents fat vacuoles. *, **, and **** represent significant difference between different groups.

The levels of TC, TG, ALT, and AST in the blood and liver of rats were measured, and the results were shown in Figure 4A–F. The results showed that a high‐fat diet significantly increased the levels of blood lipids and liver lipids in rats (*p* < 0.05). ECT intervention significantly reduced the levels of TC and TG in the rats, both in the blood and liver tissue (*p* < 0.05). Additionally, a high‐fat diet damaged the liver, significantly increasing the levels of ALT and AST in the blood (*p* < 0.05). Four weeks of ECT intervention significantly reduced liver damage caused by simple obesity (*p* < 0.05). The above results indicated that ECT had the ability to lower blood lipids and liver lipids, as well as regulate liver function.

Liver histology results revealed that the model group rats had a greater accumulation of lipid vacuoles in the liver. However, following intervention, the number of vacuoles significantly decreased, and the cell arrangement became more orderly. This suggests that a high‐fat diet increases liver fat content, while ECT exerts a favorable regulatory effect, which is consistent with the liver lipid content measurements.

### 3.5 The Regulatory Effect of ECT on Oxidative Stress Injury in Rats

**FIGURE 5:**
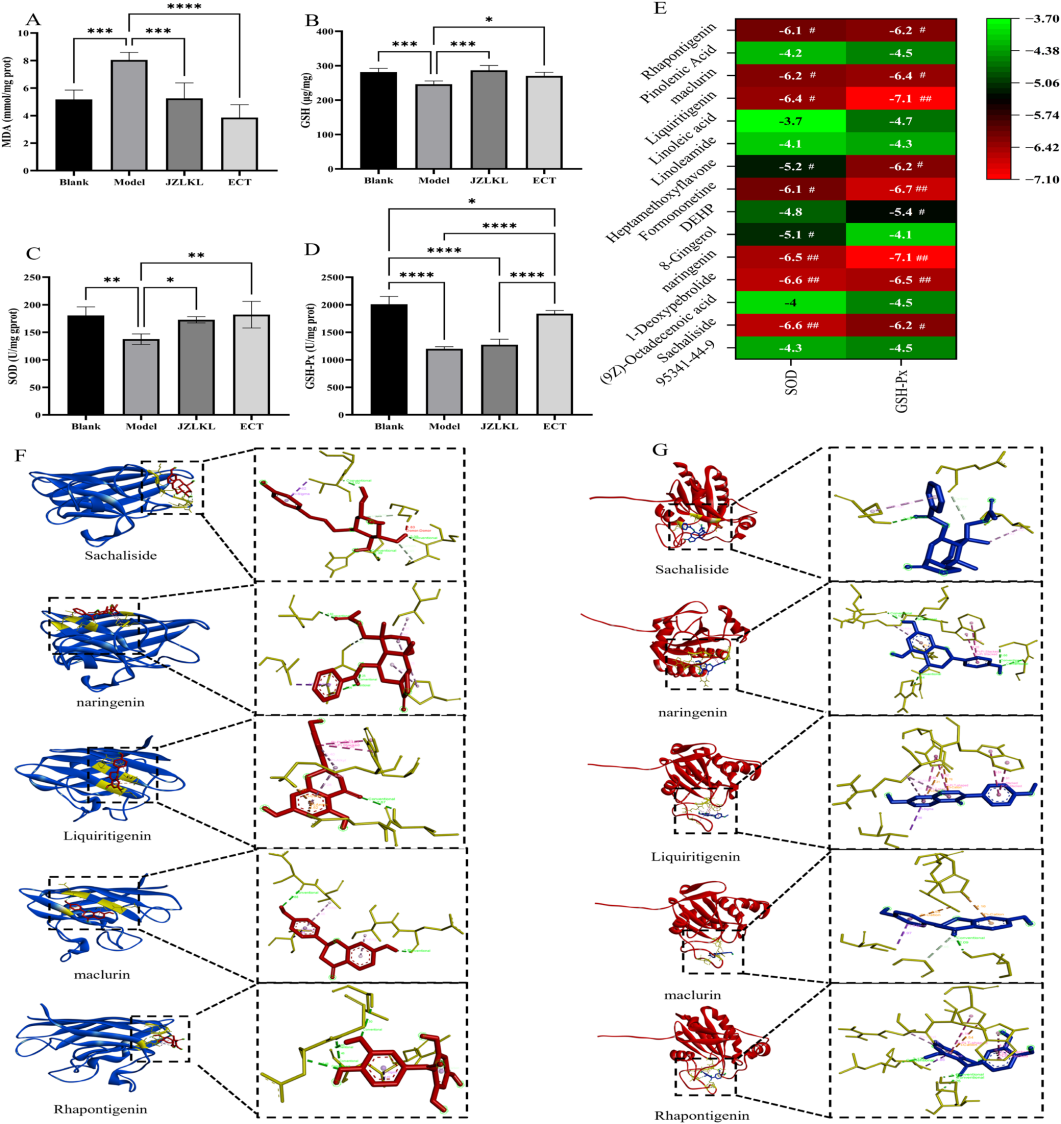
Effects of ECT on oxidative stress injury in rats. (A) Content of MDA in liver, (B) content of SOD in liver, (C) content of GSH in liver, (D) content of GSH‐Px in liver, (E) the results of molecular docking energy, (F) molecular docking of the main components of ECT with GSH‐Px, and (G) molecular docking of the main components of ECT with SOD. *, **, ***, and **** represent significant differences between different groups.

To determine the level of oxidative stress injury in rats, the contents of MDA and GSH in the liver, as well as the activities of the enzymes SOD and GSH‐Px, were measured. The results were shown in Figure 5. The results showed that the level of MDA in the model group was significantly higher than that in the control group (*p* < 0.05), while the levels of SOD, GSH, and GSH‐Px were significantly lower than those in the control group (*p* < 0.05). This indicated that obesity can lead to oxidative stress damage in rats. After the intervention, the level of MDA in the ECT group and the JZLKL group significantly reduced (*p* < 0.05), while the levels of SOD, GSH, and GSH‐Px significantly increased (*p* < 0.05). This indicated that ECT can significantly regulate oxidative stress damage caused by obesity.

In order to investigate the interaction activity between the key components of ECT and inflammatory factors, molecular docking was conducted between the top 15 components, identified through network pharmacology analysis, and SOD and GSH‐Px (Figure 5E). The results showed that Sachaliside, 1‐Deoxypebrolide, Naringenin, Liquiritigenin, and maclurin in ECT had a binding ΔG > 6.2 kJ/mol with SOD, indicating that these substances have a relatively high affinity for SOD. In ECT, 1‐Deoxypebrolide, Naringenin, Formononetine, Liquiritigenin, and maclurin had a binding energy ΔG > 6.4 kJ/mol with GSH‐Px, indicating that these substances have a relatively high affinity for GSH‐Px. These results indicated that ECT has a solid material basis for regulating oxidative stress damage in obese rats. Considering the molecular docking energy (ΔG < −7 kJ/mol indicates strong binding), the substances regulating ROS factors are Naringenin and Liquiritigenin.

### 3.6 The Regulatory Effects of ECT on the Immune System of Rats

**FIGURE 6:**
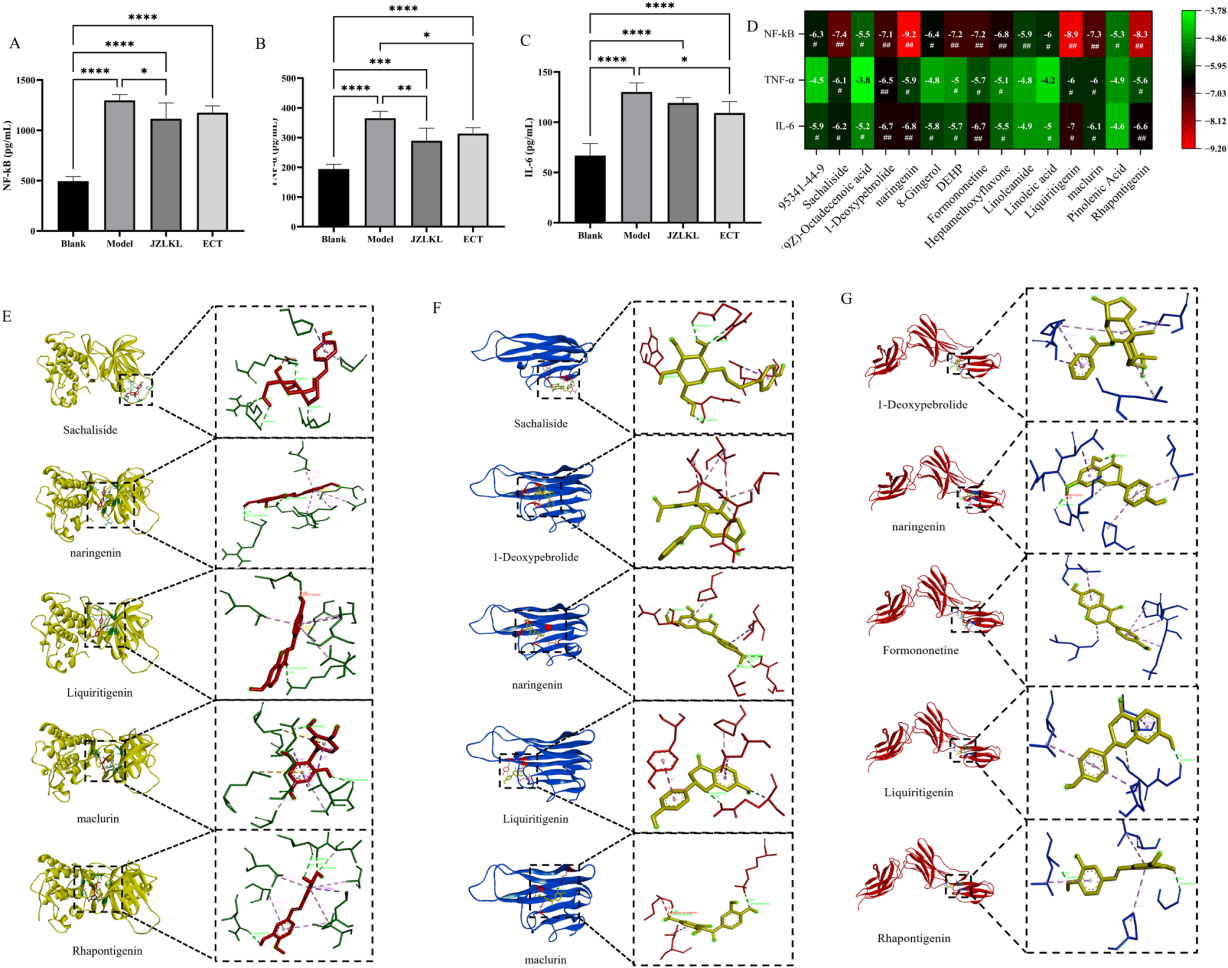
Effects of ECT on immune factors. (A) Content of NF‐κB in blood, (B) content of TNF‐α in blood, (C) content of IL‐6 in blood, (D) the results of molecular docking energy, (E) molecular docking of the main components of ECT with NF‐κB, (F) molecular docking of the main components of ECT with TNF‐α, and (G) molecular docking of the main components of ECT with IL‐6. *, **, ***, and **** represent significant differences between different groups.

To evaluate the immunoregulatory effects of ECT on obese rats, the levels of pro‐inflammatory factors NF‐κB, TNF‐α, and IL‐6 in the blood were measured. As shown in Figure 6A–C, the levels of pro‐inflammatory factors in the high‐fat diet group were significantly higher than those in the control group (*p* > 0.05), indicating that the obese rats induced by a high‐fat diet were in a state of elevated inflammation. After the ECT intervention, the levels of pro‐inflammatory factors were significantly reduced compared to the model group (*p* < 0.05), but remained higher than those in the control group. This suggested that pro‐inflammatory factors were an effective target for the regulatory effects of ECT.

In order to determine the interaction activity between the main components of ECT and inflammatory factors, molecular docking was performed between the top 15 components, ranked by degree from the network pharmacology analysis, and NF‐κB, TNF‐α, and IL‐6 (Figure 6D). The core compounds, including Sachaliside, 1‐Deoxypebrolide, Naringenin, Liquiritigenin, and maclurin, showed a higher binding ΔG with TNF‐α (ΔG > 5.9 kJ/mol). The core compounds, including Sachaliside, Naringenin, Liquiritigenin, maclurin, and Rhapontigenin, showed a higher binding ΔG with NF‐κB (ΔG > 7.3 kJ/mol). The core compounds, including 1‐Deoxypebrolide, Naringenin, Formononetine, Liquiritigenin, and Rhapontigenin, showed a higher binding ΔG with NF‐κB (ΔG > 6.6 kJ/mol). A visualization analysis was performed for the top 5 ranked binding energies, as shown in Figure 6E–G. Considering the molecular docking energy (ΔG < −7 kJ/mol indicates strong binding), the substances regulating immune factors are Sachaliside, Naringenin, Liquiritigenin, maclurin, and Rhapontigenin.

### 3.7 Pathway Prediction of ECT Regulating Obesity

**FIGURE 7:**
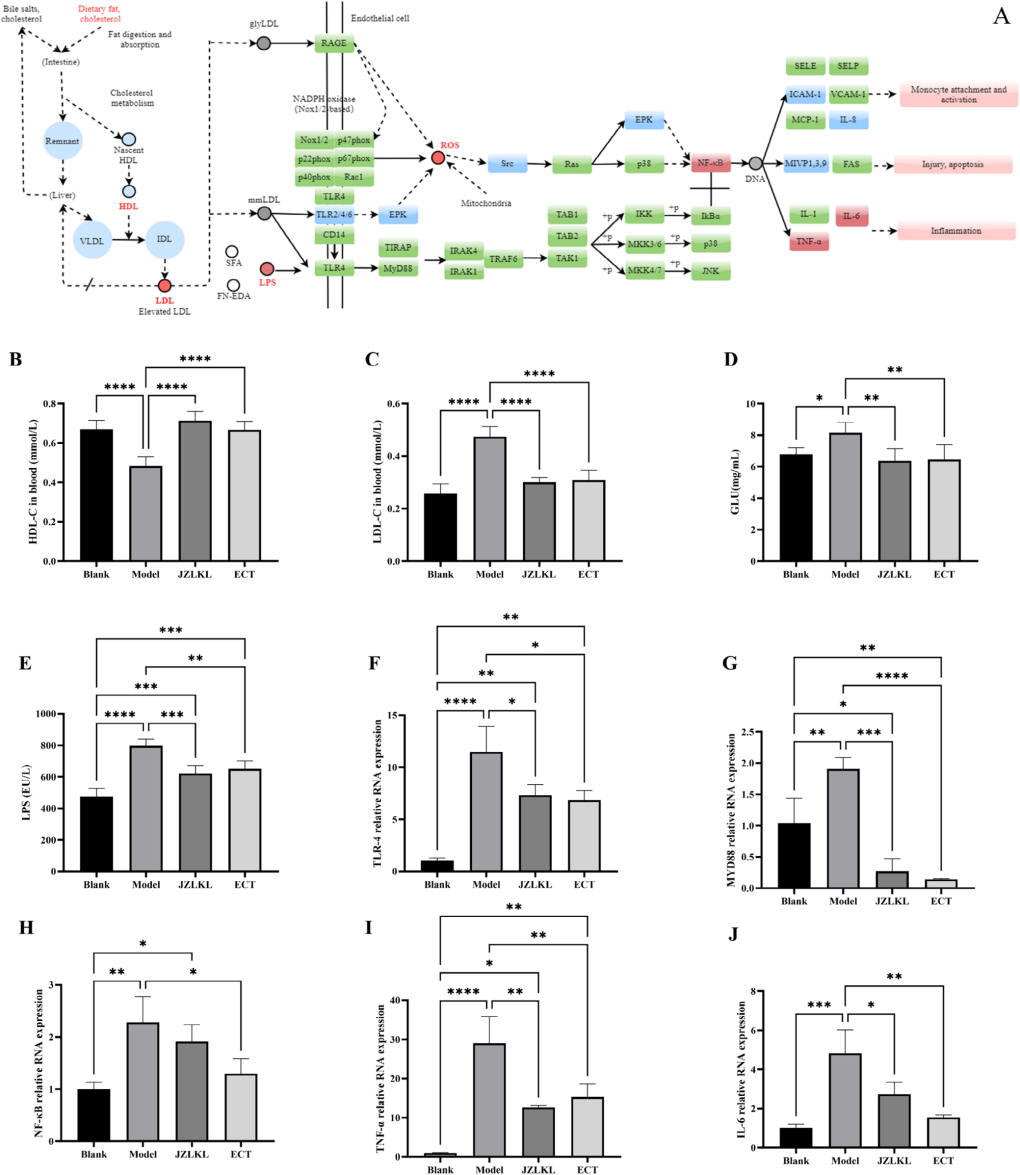
Prediction of possible pathways of ECT regulating obesity. (A) Pathway prediction map: the blue represents the enriched core targets, red represents selected validated core targets, green represents common targets. (B) Content of HDL‐C in blood. (C) Content of LDL‐C in blood. (D) Content of GLU in blood. (E) Content of LPS in blood. (F) TLR‐4 relative RNA expression. (G) MYD88 relative RNA expression. (H) NF‐κB relative RNA expression. (I) TNF‐α relative RNA expression. (H) IL‐6 relative RNA expression. *, **, ***, and **** represent significant difference between different groups.

KEGG enrichment analysis of the core ECT‐obesity targets predicted potential pathways through which ECT may regulate obesity, as shown in Figure 7. HDL, LDL, and LPS are key factors contributing to oxidative stress and inflammatory damage induced by obesity. In the intestinal epithelium, TLR4 serves as the primary receptor for these molecules. Reducing the production of LDL and LPS, along with inhibiting TLR4 overexpression, can downregulate MyD88 expression, thereby alleviating inflammatory responses. Additionally, ECT may mitigate oxidative stress‐induced inflammation via the ERK pathway. These mechanisms highlight the crucial role of ECT in reducing obesity‐induced oxidative damage and inflammatory responses. Under hyperglycemic conditions, LDL can form glyLDL, which stimulates the production of ROS, activating oxidative stress.

To preliminarily verify the possibility of this pathway, the levels of HDL‐C (Figure 7B), LDL‐C (Figure 7C), and LPS (Figure 7D) in the blood were measured. As expected, the levels of LDL‐C, LPS, and GLU in the model group were significantly higher than those in the blank group (*p* < 0.05), while the HDL‐C level was significantly lower than that of the blank group (Figure 7C) (*p* < 0.05). After 4 weeks of intragastric administration of ECT, the levels of LDL‐C, LPS, and GLU in the blood were significantly reduced, while the HDL‐C level was significantly increased (*p* < 0.05).

The relative RNA expression of TLR‐4 and MYD88 in the model group was higher than in the blank group (*p* < 0.05). It was significantly reduced after a 4‐week intervention (*p* < 0.05) and there was no significant difference between JZLKL and ECT. In addition, the levels of immune factor expression, including NF‐κB, TNF‐α and IL‐6, were reduced. This suggests that ECT may modulate simple obesity through the TLR4/MyD88/NF‐κB signaling pathway. This suggests that ECT has the potential to significantly alleviate obesity‐associated inflammation. These results indicated that the weight loss, lipid‐lowering effects, and obesity‐induced inflammation alleviation mechanisms of ECT are consistent with the predicted regulatory pathways shown in Figure 7A.

## 4 Discussion

The study determined the key components of ECT using UHPLC‐OE‐MS/MS technology and explored its potential targets through network pharmacology. Then, we validated its weight loss and lipid‐lowering effects through animal experiments. By measuring liver oxidative stress levels, serum inflammatory factor levels, as well as lipid and LPS levels in the serum, combined with molecular docking experiments of the main core components, the study further clarified the material basis of ECT in regulating obesity and found possible regulatory pathways (TLR4/MyD88/NF‐κB) of ECT. The results suggested that ECT holds potential for development as a functional food in the future.

Network pharmacology serves as an important approach for investigating the therapeutic and regulatory effects of traditional herbal formulas on various diseases (Zhao et al. 2023). However, current studies primarily rely on database‐based predictions of active compounds and their targets, as exemplified by network pharmacology analyses of Gualou Xiebai Banxia Decoction for myocardial infarction (Wang and Chen 2024) and Albiziae Flos for depression (Xiong et al. 2020). However, variations in preparation methods often lead to differences in the types and concentrations of compounds. Experimental determination can effectively mitigate this risk, enhancing the accuracy and reproducibility of the research findings. This study identified 93 major compounds in ECT, mainly including polyphenols, flavonoids, terpenoids, organic acids, and polysaccharides. Molecular docking analysis revealed that the key bioactive compounds Sachaliside, Naringenin, Liquiritigenin, maclurin, and Rhapontigenin, which play crucial roles in regulating oxidative stress and modulating immune responses, are likely derived from *Pinellia ternata* (Sachaliside).

*Citrus reticulata* (Naringenin), *Glycyrrhiza glabra* L. (Liquiritigenin), *Prunus mume* (maclurin), and either *Poria cocos* or *Prunus mume* (Rhapontigenin), respectively. Previous studies have shown that *Pinellia ternata* contains alkaloids and organic acids, among other compounds (Xue et al. 2021). The key components of *Citrus reticulata* (chenpi) were hesperidin, nobiletin, and tangeretin, which were primarily flavonoid compounds (Ke et al. 2020). They had a beneficial regulatory effect on liver steatosis and oxidative stress in rats. The polysaccharides and triterpenoids in *Poria cocos* were its main compounds (Rios 2011), and they can reduce atherosclerosis by inhibiting inflammation (Li et al. 2021). Ginger was rich in natural polyphenols, such as gingerols, which have strong anti‐inflammatory effects (Pazmandi et al. 2024). *Prunus mume* was rich in various phenolic and polysaccharide compounds, and possessed strong antioxidant properties (Kumari and Gunathilake 2020).

Further analysis of the active components of ECT was conducted using network pharmacology methods to elucidate its potential pharmacological mechanisms. A total of 746 targets were obtained, of which 221 were obesity‐related targets. In addition, 270 BP (biological processes), 35 CC (cellular components), 51 MF (molecular functions), and 185 KEGG‐related signaling pathways were involved. KEGG enrichment analysis results showed that the signaling pathways involved in ECT intervention in obesity mainly include the regulation of lipolysis, the TNF‐α inflammatory pathway, PPAR, and insulin resistance pathways. Previous studies have shown that pro‐inflammatory factors TNF‐α, IL‐6, and IL‐12 in obese individuals can trigger inflammation and insulin resistance (Wager and Wormley Jr. 2014). According to the pathway prediction from KEGG, LDL and LPS were the triggering substances in the ECT‐regulated obesity pathway, with LDL being formed through liver metabolism after a high‐fat diet. LPS may enter the bloodstream due to increased intestinal permeability. This study confirmed that ECT might alleviate obesity by reducing LDL and LPS levels in obese rats.

When total energy expenditure remains unchanged, feeding a high‐fat diet increases the body's energy intake, thereby burdening lipid metabolism in the liver and raising TC and TG levels in the blood and liver. Over time, the amount and size of adipose tissue increase, leading to weight gain and a higher liver index. In severe cases, liver damage happened (Milic et al. 2014). These findings were consistent with the results obtained in this study. ECT intervention significantly reduced body weight, liver index, epididymal fat index, subcutaneous fat index, blood lipid, and liver lipid levels. Additionally, the levels of AST and ALT, which indicated the degree of liver damage, were also significantly reduced (*p* < 0.05). In addition, ECT significantly reduced food intake in obese rats and decreased the food efficiency ratio (*p* < 0.05). This effect may be attributed to ECT's ability to suppress the expression of appetite‐related factors and inhibit the activity of digestive enzymes in rats, thereby reducing the absorption and conversion of energy substances. However, this mechanism was not explored in detail in the present study.

Under hyperglycemic conditions, LDL undergoes glycation to form glyLDL. glyLDL can promote the production of ROS in cells or activate NADPH oxidase, thereby exacerbating oxidative stress (Figure 7A). In this study, blood glucose and LDL levels were significantly elevated in the model group (*p* < 0.05), promoting the production of glyLDL, which in turn exacerbated oxidative stress damage in obese rats (Figure 7B–E). The intervention of ECT significantly reduced the levels of the oxidative stress marker MDA in the liver of rats, activated the enzymatic activities of SOD and GSH‐Px, and alleviated oxidative stress damage induced by simple obesity (Figure 5). Molecular docking revealed that Sachaliside, 1‐Deoxypebrolide, Naringenin, Liquiritigenin, and maclurin in ECT exhibited the strongest binding affinities for SOD, while 1‐Deoxypebrolide, Naringenin, Formononetine, Liquiritigenin, and maclurin showed strong binding affinities for GSH‐Px. These findings indicated that the polyphenols Naringenin and Liquiritigenin are the primary contributors to the oxidative stress‐modulating activity of ECT. Previous studies have shown that Naringenin exerts antioxidant effects by donating hydrogen atoms or electrons to neutralize free radicals, thereby interrupting the propagation of radical chain reactions (Cai et al. 2019). The antioxidant activity of Liquiritigenin mainly relies on its phenolic hydroxyl group at the C4′ position, which scavenges reactive oxygen species through multiple coordinated mechanisms, including hydrogen atom transfer, electron transfer, and proton transfer (Ma and Wang 2023). Previous studies have shown that Liquiritigenin, the main polyphenolic component of licorice, possesses strong antioxidant capacity (Ma and Wang 2023). Naringenin, derived from *Citrus reticulata* , also exhibited strong antioxidant activity (Zhang et al. 2022). The plant‐derived polyphenol maclurin can effectively prevent oxidative stress damage induced by hydroxyl radicals (Li et al. 2014).

When oxidative stress was activated in the body, it triggers the activation of p38 and ERK proteins in the MAPK pathway, which in turn activates NF‐κB, leading to the promotion of pro‐inflammatory factors such as IL‐6 and TNF‐α (An et al. 2019). In addition, the increase in LPS would stimulate the expression of the TLR4 protein, and their binding would activate the NF‐κB signaling pathway via the MyD88 adaptor. Together, they promote the occurrence of the inflammatory response. In our study, we found ECT could regulate obesity via the LPS‐TLR4/MyD88/NF‐κB signaling pathway via RT‐qPCR and Elisa experiments. However, the ROS pathway mediated by the EPK signaling cascade was not fully assessed. Nevertheless, our findings clearly demonstrated the regulatory effect of ECT on ROS levels. The results of network molecular pharmacology indicated that IL‐6 and TNF‐α were key targets for ECT in regulating obesity. Animal experiments also demonstrated that ECT significantly downregulated IL‐6, TNF‐α, and NF‐κB. Through molecular docking experiments, it was found that the core components Naringenin, Liquiritigenin, and maclurin exhibited high binding affinities to the three pro‐inflammatory factors. Existing studies have shown that Naringenin and Liquiritigenin can downregulate the NF‐κB pathway, demonstrating significant anti‐inflammatory potential (Kim et al. 2008; Wang et al. 2020). The anti‐inflammatory effects of maclurin have been confirmed both in vitro and in vivo, but its specific regulatory pathways have yet to be identified (Lee et al. 2024). Therefore, these compounds provided the material basis for ECT‐mediated regulation of obesity through the TLR4/MyD88/NF‐κB signaling pathway.

Although this study demonstrated the lipid‐lowering and anti‐inflammatory effects of ECT, several limitations remain. The dose–response relationship has not been systematically evaluated, and gradient dosing studies are lacking. Additionally, as a multi‐component formulation, the interactions and pharmacokinetics of ECT's active compounds are not fully understood, which limits its standardization and development as a functional food.

## 5 Conclusion

Through component analysis, network pharmacology, and animal experiments, we predicted and validated the potential of ECT for weight loss and lipid reduction. Furthermore, we identified the key bioactive compounds in ECT (Sachaliside, Naringenin, Liquiritigenin, maclurin, and Rhapontigenin) and elucidated its underlying LPS‐TLR4/MyD88/NF‐κB signaling pathway in mitigating obesity‐induced oxidative stress and inflammatory responses. These findings suggest that ECT holds promise for further development as a functional food for the regulation of simple obesity.

## Author Contributions

**Longfei Zhang:** data curation (equal), formal analysis (equal), investigation (equal), methodology (equal), software (equal), visualization (equal), writing – original draft (equal), writing – review and editing (equal). **Xiaoxiao Liu:** data curation (equal), formal analysis (equal), funding acquisition (equal), supervision (equal), writing – original draft (equal), writing – review and editing (equal). **Mingze Xu:** data curation (equal), project administration (equal). **Yang Liu:** visualization (equal). **Jiahao Yang:** methodology (equal). **Wenqiong Wang:** resources (equal). **Hengxian Qu:** software (equal). **Tianzhu Guan:** project administration (equal), resources (equal). **Dawei Chen:** funding acquisition (equal), validation (equal). **Ruixia Gu:** conceptualization (equal), funding acquisition (equal), investigation (equal), visualization (equal).

## Conflicts of Interest

The authors declare no conflicts of interest.

## Supporting information


**Figure S1.** The TIC chromatograms in both positive and negative ion modes.

## Data Availability

We declare that we have no financial or personal relationships with other people or organizations that can inappropriately influence our work; there is no professional or other personal interest of any nature or kind in any product, service, and/or company that could be construed as influencing the position presented in, or the review of, the manuscript entitled.
