# Supplementary figures and images for: Erchen Decoction Offers Health Benefits in Regulating Obesity: Component Analysis, Network Pharmacology, and Experiment Verification
Source: Food Sci Nutr. 2025 Aug 5;13(8):e70592. doi: 10.1002/fsn3.70592 (PMC12325893; doi:10.1002/fsn3.70592)

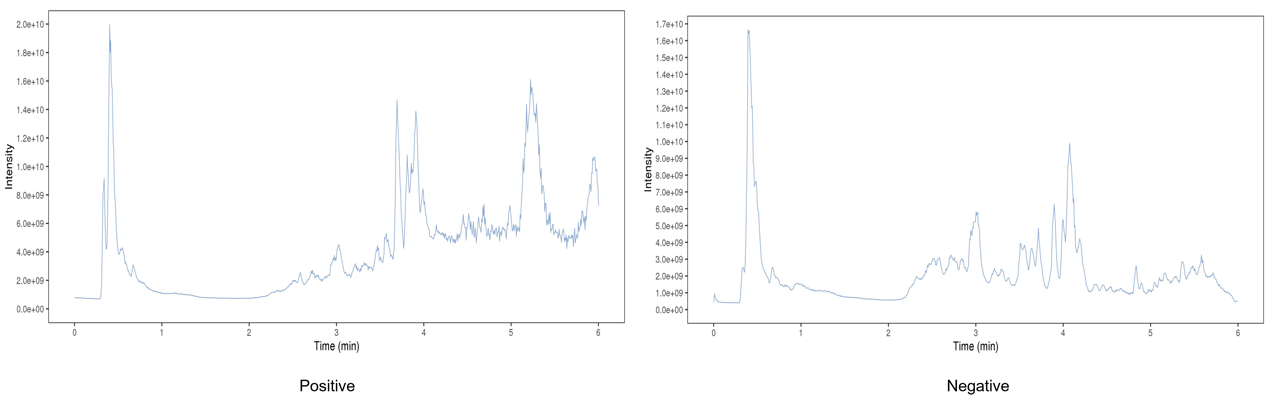


Figure S1 The TIC chromatograms in both positive and negative ion modes.

Supplement: Supplementary file 1 — Figure S1. The TIC chromatograms in both positive and negative ion modes. [file FSN3-13-e70592-s001.docx]
